# 

**Published:** 1859-10

**Authors:** 


### Contents of the Chicago Medical Journal---October, 1859.

ORIGINAL COMMUNICATIONS. PAGE.

ART. I.—Placental Presentation. By Wm. H. Baltzell, M. D., of Chicago ........ 581
ART. II.—Expectoration of Fibrinous Concretions, or "False Membrane," from the Bronchiæ of an Adult. By L. H. Angell, M. D., of Aurora, Ill........ 591
ART. III.—Diphtheria. By W. Godfrey Dyas, M. D., Chicago........ 595

SELECTIONS.

On the Procreation of Male or Female Children, at will. Presented by John E. Van Molle, A.M., of Brussels, to the Faculty of Oglethorpe College of Savannah, for admission to the grade of Doctor in Medicine. (From the *Oglethorpe Medical and Surgical Journal.*) 606
On the Causes of Death after Amputation. By Mr. Bryant, Assistant Surgeon to Guy's Hospital. (Proc. of the Roy. Med. and Chir. Soc., Feb. 22, 1859.)........ 609
Cursory Remarks on the Diagnosis of Fatty Heart. By Henry Kennedy, A.B., M.B., Fellow and Censor of the College of Physicians in Ireland, Physician Extraordinary to Sir P. Dun's Hospital. (Read before the Medical Association, June 1, 1859.) (From the *North American Medico-Chir. Review.*)........ 612
Late Researches on the Atmosphere. (From the *New Orleans Medical and Surgical Journal.*) 623
Clinical Records. (From the *London Lancet.*)—
- Tonics and Iron in Erysipelas of the Face and Scalp........ 625
- Excision of the Knee-Joint for Old Standing Disease........ 625
- Disease of the Sternum simulating Aneurism........ 626
- Tobacco-Pipe Stem in the Throat........ 628
- Encephaloid Disease of the Epididymis........ 628
- Nasal Carcinoma........ 629
- Useful Plan of Supporting Stumps after Amputation........ 629
- Tumor of the Parotid........ 630
- Double Fistula in Ano, treated by a Single Division of the Sphincter........ 631

BOOK AND PAMPHLET NOTICES.

A Practical Treatise on Enteric Fever; its Diagnosis and Treatment: being an Analysis of One Hundred and Thirty Consecutive Cases, derived from Private Practice, and embracing a Partial History of the Disease in Virginia. By James E. Reeves, M.D. Philadelphia: J. B. Lippincott & Co. 1859........ 632
The Dental Cosmos........ 637

EDITORIAL.

Notes of Surgical Operations.
1. Foreign Body in the Urinary Bladder........ 638
2. Extirpation of the Globe of the Eye for Cancer........ 638
3. Trephining for Epilepsy and Insanity........ 639
4. Reduction of Dislocations of the Hip by Manipulation........ 641

Prospectus of the Chicago Medical Journal for 1860........ 642
Obituary........ 643

